# Inflammatory Mediators Release in Urine from Mice Injected with *Crotalus durissus terrificus* Venom

**DOI:** 10.1155/2011/103193

**Published:** 2011-11-29

**Authors:** A. Hernández Cruz, L. Barbosa Navarro, R. Z. Mendonça, V. L. Petricevich

**Affiliations:** ^1^Facultad de Medicina, Universidad Autónoma del Estado de Morelos (UAEM), Calle Iztaccihuatl Esquina Leñeros, Colonia Volcanes, 62350 Cuernavaca, MOR, Mexico; ^2^Laboratório de Parasitologia, Instituto Butantan, Avenida Vital Brasil 1500, 05555 São Paulo, SP, Brazil

## Abstract

In this study, we investigated in groups of female BALB/c mice injected with *Crotalus durissus terrificus* venom (*Cdt*) the renal function based on creatinine clearance, percentage of fractional excretion cytokines and histological examination of renal tissue. *Cdt* caused renal alterations that induced proteinuria during the initial hours post-venom and reduced creatinine clearance 15 min. up to 2 hours post-venom administration. In urine from mice injected with *Cdt* induced a decrease in IL-4 levels. More pronounced increments of IL-5, IL-6 and IFN-**γ** were observed after 15 and 30 min, respectively. The highest levels of TNF and IL-10 were observed at 1 and 4 hs, respectively. The ratios of pro- and anti-inflammatory cytokines in animals injected with *Cdt*, which may be manifested in the inflammatory status during the envenoming. In groups of animals treated with *Cdt* were observed a decreasing in creatinine clearance and its effect on glomerular filtration rate was accompanied by decreased fractional excretion of cytokines and morphologic disturbances. This loss of change selectively in envenomation could thus explain why the relatively excretion of cytokines is reduced while of total proteins increases. In conclusion the fractional excretion of cytokines is significantly reduced in mice injected with *Cdt*, despite proteinuria.

## 1. Introduction

Snake venom presents biological actions, chemical composition, toxicity, and pharmacokinetics and pharmacodynamic characteristics and is a mixture of complex toxins such as neurotoxins, myotoxins, coagulations, nephrotoxins, and necrotoxins. It is important to understand that the actual mix of toxins in the venom vary well by individual species and also by age and season [1]. The Brazilian *Crotalus durissus terrificus *snake is responsible for many clinical cases of envenoming. Its venom contains a variety of toxic proteins including crotoxin, crotamine, gyroxin, convulsin, and a thrombin-like enzymes [2, 3]. Envenomation caused by *Crotalus *snake leads to serious complications and alterations such as systemic hemorrhage, hepatotoxicity, myotoxicity, hypotension, acute renal failure, and shock [4–7]. It has been related to myolysis, hemolysis, hypotension, and/or direct venom nephrotoxicity [7]. This venom has myotoxic activity which leads to the development of rhabdomyolysis that can be followed by skeletal muscle damage and the release of creatinine phosphokinases, lactate dehydrogenase, and myoglobin [8]. The pathogenesis of acute renal failure after snake bites appears to be multifactorial such as bleeding, hypotension, circulatory collapse, intravascular hemolysis, dissemination intravascular coagulation, and also direct nephrotoxicity of the venom [8, 9]. These effects are defined as deterioration of renal function manifested by elevation in blood urea nitrogen and serum creatinine, disturbances in electrolyte and acid-base homeostasis, and retention of nitrogenous waste products, occurring in hours or to days and/or to weeks [9]. Acute renal failure is a frequent complication observed in victims of snakebites. Evaluation of renal function parameters after crude venom administration has suggested a direct effect on glomerular filtration rate, a slow and steady accumulation of nitrogenous waste products, and an inability of the kidney to regulate the balance of sodium, electrolytes, acid, and water [10–13]. Girón et al. [14] demonstrated that the intravenous and intraperitoneal administration of *C. vegrandis* crude venom induce damage that encompasses the area of proximal tubules and peritubular vessels and damage to the glomerular capillary endothelial cells as target of its action in absence of hemoglobin. 

The kidney is the organ to help remove toxins, due to its high blood flow and its capacity to concentrate substance in urine. Numerous *in vitro* and *in vivo* studies about renal inflammation have been accompanied by a better understanding of the phenotypic changes and multifunctional potentials of local and infiltrating cells within the site of inflammation. These include activation, transition, plasticity, adhesion, trafficking of residential cells, and infiltrating inflammatory cells such as neutrophils, monocytes/macrophages, and lymphocytes. These cells release inflammatory mediators, which include different sets of adhesion molecules, proteases and degrading enzymes, oxygen free radicals, nitric oxide, chemokines, growth factors, and different types of cytokines. Clinical and experimental animal studies reveal that renal inflammation can be modulated by inflammatory mediators [15, 16]. 

The purpose of the present study was to determine the urinary cytokine levels in groups of mice treated with different amounts of *Crotalus durissus terrificus* venom and to examine the role of IL-6, IL-10, and TNF-*α* in the pathogenesis of venom-induced acute renal failure. The production of proinflammatory cytokines such as IL-6 and TNF-*α* is responsible for initiation of an effect against exogenous stimulus. However, excessive production of these mediators may significantly contribute to shock, multiple organ failure, and death [17–21]. In contrast, anti-inflammatory cytokines such as IL-4 and IL-10 are crucial for downregulating the increment inflammatory process and maintaining homeostasis for the correct functioning of vital organs [21, 22]. 

## 2. Material and Methods

### 2.1. Chemicals, Reagents, and Buffers

Actinomycin D, orthophenyldiamine (OPD), fetal calf serum (FCS), and RPMI-1640 medium were purchased from Sigma (St. Louis, Mo, USA), murine anti-IL-4 (clones 11B11 and BVD6-24G2) recombinant IL-4, murine anti-IL-5 (clones TRFK5 and TRFK4), recombinant IL-5, murine anti-IL-6 (clones MP5-20F3 and MP5-32C11), recombinant IL-6, murine anti-IL-10 (clones JES5-16E3 and SXC-1), recombinant IL-10 and murine anti-IFN-*γ* (clones XGM1.2 and R4-6A2), and recombinant IFN-*γ* were purchased from BD Biosciences Pharmingen (USA), and recombinant TNF was purchased from Boehringer Mannheim (Mannheim, Germany).

### 2.2. Venom

Lyophilized venom of *Crotalus durissus terrificus* (*Cdt*) was obtained from the Laboratory of Herpetology, Instituto Butantan, SP, Brazil and stored at −20°C. The venom was dissolved in sterile physiological saline (0.85% (w/v) NaCl solution) immediately before use.

### 2.3. Animals

Females BALB/c mice (6–8 weeks old, weighing 13 to 15 g) were maintained in Bioterio-Facultad de Medicina, UAEM (Cuernavaca, México). The animals were maintained and used under strict ethical conditions according to international recommendations for animal welfare the Committee Members, International Society in Toxicology [23].

### 2.4. Lethality Assay

Probits method was used to calculate the lethal dose fifty (LD_50_) of the *Cdt *venom. The mice weighing 13 to 15 g were intraperitoneally (i.p.) injected with 0.1 mL of various concentrations of venom, and the number of mice died was counted after 48 h. The number of mice used at each dose was four [24]. 

### 2.5. Blood and Urine Biochemical

#### 2.5.1. Blood Collection

Groups of female BALB/c mice with 13–15 g were injected intraperitoneously (i.p.) with different amounts of *Cdt *dissolved in 0.1 mL of saline solution. Control mice received 0.1 mL of saline solution. Different times after injection with *Cdt,* animals were bled by retro-orbital plexus. The blood samples were allowed to stand until they formed a clot, and the sera were used in biochemical analysis [24].

#### 2.5.2. Urine Collection

Mice were placed in metabolic cages with ample water but no solid food. The animals were intraperitoneally injected with different doses of *Cdt* venom, and the urine was collected in a recovery tube on ice. Mice produced an average of 0.05–0.100 mL of urine/mouse during different times. After collection their urine was spun to remove any solid debris and was stored at −70°C until batch ELISA cytokine measurements were performed. 

#### 2.5.3. Creatinine Levels

Present in sera and urine from control mice or injected with different amounts of *Cdt* venom were determined using specific kits (Spinreact diagnostic, Sant Esteve de Bas, Spain), according to the manufacture's protocol [24].

#### 2.5.4. Creatinine Clearance

Renal clearance (*C*) was calculated using the standard formula:  *C* = (*U* × *V*)/*S*(*μ*L/min/100 g) where *U* is urinary concentration, *V* is urinary volume/min, and *S* is the serum creatinine levels for each point.

#### 2.5.5. Percentage of Fractional Excretions

Indicator clearance rations were calculated using the formula: (Urine_cre_/Serum_*X*_ : Urine_*X*_/Serum_cre_) × 100, where *X* is cytokine concentration (TNF, IL-6, IL-10, IL-4, and IFN-*γ*).

### 2.6. Proteins Assay

Total urine protein excretion was determined as previously described by Timoshanko and Tipping [25]. In brief, mice were housed individually in cages to collect urine before administration of *Cdt*, and spontaneously voided urine was collected over different times. Urine samples were diluted in 1% 5-sulfosalicylic acid, and the protein concentrations were determined spectrometrically at a wave length of 550 nm and compared with known albumin standards.

### 2.7. Mediators's Production

#### 2.7.1. Cytokines

The levels of cytokines IL-4, IL-5, IL-6, IL-10, and IFN-*γ* present in urine from BALB/c mice were assayed by a two-site sandwich enzyme-like immunosorbent assay (ELISA) [26]. Briefly, ELISA plates were coated with 100 *μ*L (1 *μ*g/mL) of the monoclonal antibodies anti-IL-6, anti-IL-10, or anti-IFN-*γ* in 0.1 M sodium carbonate buffer (pH 8.2) and incubated for 6 h at room temperature. The wells were then washed with 0.1% phosphate-buffered saline (PBS/Tween-20) and blocked with 100 *μ*L of 10% FCS in PBS for 2 h at room temperature. After washing, duplicate urine samples of 50 *μ*L were added to each well. After 18 h of incubation at 4°C, the wells were washed and incubated with 100 *μ*L (2 *μ*g/mL) of the biotinylated monoclonal antibodies anti-IL-4, anti-IL-5, anti-IL-6, anti-IL-10, or anti-IFN-*γ* as second antibodies for 45 min at room temperature. After a final wash, the reaction was developed by the addition of OPD to each well. Optical densities were measured at 405 nm in a microplate reader. The cytokine content of each sample was read from a standard curve established with the appropriate recombinant cytokines (expressed in nanograms per milliliter). The minimum levels of each cytokine detectable in the conditions of the assays were 10 pg/mL for IFN-*γ*, IL-4, and IL-5 and 5 pg/mL for IL-10 and IL-6.

To measure the cytotoxicity of TNF present in urine samples, a standard assay with L929 cells, a fibroblast continuous cell line, was used as described previously by Ruff and Gifford [27]. The percentage cytotoxicity was calculated as follows: (*A*_control_ − *A*_sample_/*A*_control_) × 100. Titers were calculated as the reciprocal of the dilution of the sample in which 50% of the cells in the monolayers were lysed. TNF activity is expressed as pg/mL estimated from the ratio of a 50% cytotoxic dose of the test to that of standard mouse recombinant TNF.

### 2.8. Histopathological Assay

After treatment both right and left kidneys were fixed in 10% paraformaldehyde/PBS (pH 7.4). The organs were embedded in paraffin, and sections with thickness 4 *μ*m were cut by rotary microtome. The slides were stained with hematoxylin and eosin (H & E) and were examined with a light microscope using a 1 × 100 or 1 × 400 lens. Images were captured with a coupled-device camera and exported to Adobe Photoshop 7.0.

### 2.9. Statistical Analyzes

Data are expressed as the mean ± standard deviation. Statistical analyses were performed by Student's *t*-test, and the level of significance was set at *P* < 0.05.

## 3. Results

The effects of different doses of *Crotalus durissus terrificus *venom were analyzed by detecting the mortality and the levels of proteins, creatinine, and cytokines in urine from BALB/c female mice with 13–15 g of body weight.

### 3.1. Effect of Cdt on Lethality

We previously described that 1 LD_50_ for female BALB/c mice with 13–15 g body weight corresponds to 0.01 mg/kg (data not shown). For groups of animals that received 0.5 and 1 LD_50_ of *Cdt*, the mortality was 0% and 50%, respectively, (data not shown). In contrast, for the groups of mice injected with 2 and 2.5 LD_50_ of *Cdt* the mortality observed was 90% and 100%, respectively, (data not shown). Death was usually preceded by certain signals, or symptoms were observed, and the highest mortality percentage observed for groups of mice injected with 1 to 2.5 LD_50_ was between 4–6 hs (data not shown).

### 3.2. Effect of Different LD_50_ of Cdt on Urine Protein, Creatinine Clearance, and Cytokines Production

**Figure 1:**
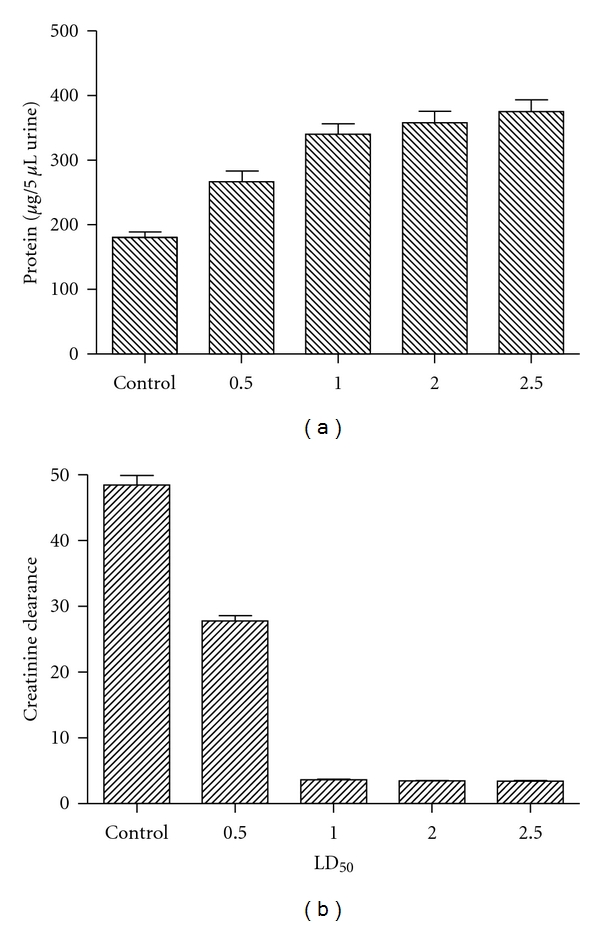
Protein present in urine and creatinine clearance. Groups of BALB/c female mice were injected i.p. with different amounts of *Cdt* for different times as above described. The presence of protein in urine was determined by measuring the albumin. Creatinine levels were detected in urine and serum from mice injected with different amounts of *Cdt* for 1 h. The formula to determine creatinine clearance is described in Section 2. Each bar represents the mean value of samples from four experiments in different groups of five mice. Statistical differences between the treatments were (*P* > 0.01).

**Figure 2:**
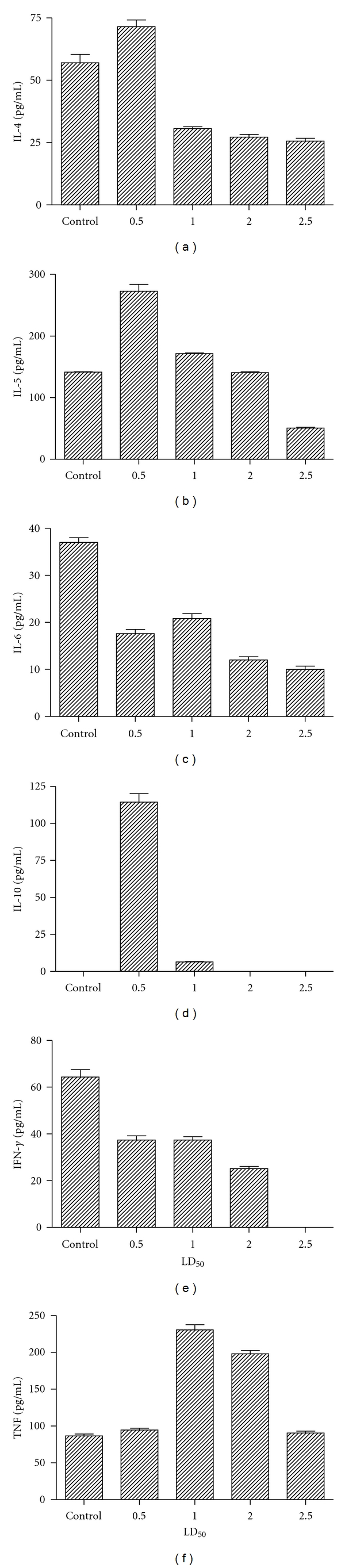
Cytokine excreted in urine from BALB/c female mice. Urine of mice was obtained after i.p. injection with different amounts of *Cdt* as above described. The levels of IL-5 were determined after 15 min, for IL-4, IL-6, IL-10, and IFN-*γ* after 1 h. They were assayed by ELISA assay using monoclonal antibodies as the probe. TNF levels were determined after 1 h by standard assay with L929 cells. Each bar represents the mean value of samples from four experiments in different groups of five mice. Statistical differences between the treatments were (*P* > 0.01).

In order to determine the levels of protein, creatinine clearance, and cytokines present in urine, groups of mice were injected with different LD_50_ of *Cdt* venom for distinct times. As shown in Figure 1, the levels of protein present in urine from mice treated with *Cdt *were significantly higher when compared with those obtained in urine control mice, and these increments were dose dependent (Figure 1). Creatinine clearance observed in groups of mice treated with different amounts of *Cdt* were significantly lower when compared with those obtained in the control group (Figure 1). The ability of *Cdt* to induce the production of cytokines levels is shown in Figure 2. Among groups of mice treated with 0.5 LD_50_ of *Cdt*, the urine levels of IL-4, IL-5, and IL-10 were significantly higher when compared with the control groups (*P* < 0.001). The urine levels of IL-6 and IFN-*γ* present from mice i.p. administrated with different LD_50_ of *Cdt* were significantly lower than those obtained in urine from control groups (Figure 2). The decrease observed for these two cytokines was dose-dependent. The highest levels of TNF were observed for urine from mice injected with 1 and 2 LD_50_ (Figure 2). Also was possible observing that with increment of venom concentrations the levels of IL-4, IL-5, IL-6, IL-10, and IFN-*γ* were significantly lower when compared with those obtained in urine from control groups (*P* < 0.0001). 

### 3.3. Effect of Cdt on Glomerular Filtration Rate

Taken these results, it was possible to establish the evolution of inflammatory and renal injury in mice treated with *Cdt*. Thus, the following set of experiments to determine the effect of *Cdt* on renal function groups of BALB/c female mice with 13–15 g of body weight were injected with 1 LD_50_ of *Cdt* for different time periods, and the urine was collected. 

The time course of the presence of protein in urine from mice injected with 1 LD_50_ of *Cdt* is shown in Table 1. The amounts of protein started to appear after 15 min in groups of mice i.p. injected with *Cdt*. The maximum amounts of protein were observed in urine from mice injected with *Cdt* for 1 h, decaying thereafter. At this time, the amounts of protein present in urine from mice injected with *Cdt* were significantly different when we compared those obtained in urine from control groups (*P* < 0.005). However, the level of proteinuria had recovered considerably after 48 hs. The levels of creatinine started to increment 15 min in groups of mice injected with 1 LD_50_ of *Cdt*. Creatinine levels in urine from mice injected with *Cdt* venom were significantly higher when compared with those obtained in control group (data not shown). As shown in Table 1, the progressive decrease in renal creatinine clearance was maximal 1-2 hs postvenom injection with return to basal levels after 48 hs. 

**Figure 3:**
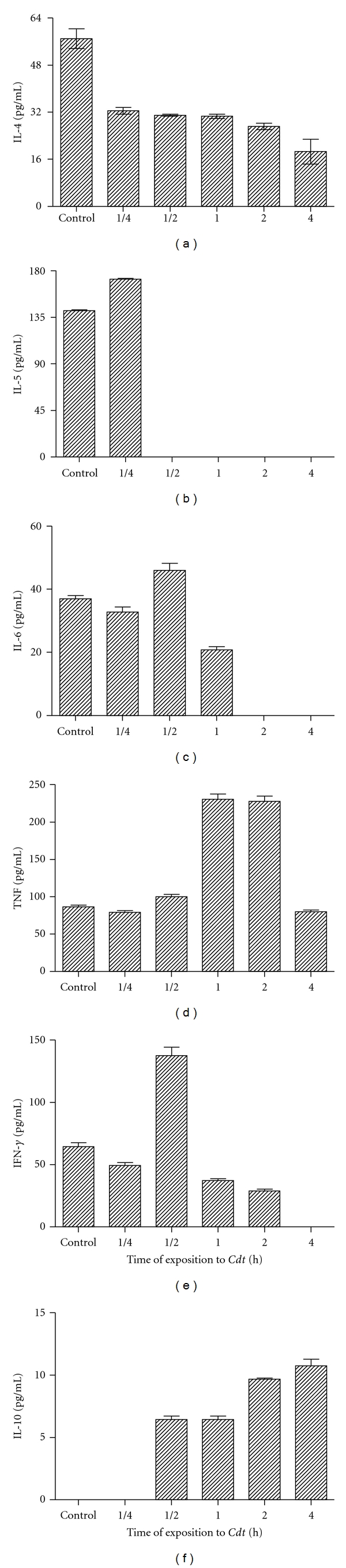
Kinetics of cytokines. Groups of BALB/c female mice were injected i.p. with 1 LD_50_ of *Cdt* for different times as above described. The levels of cytokines excreted in urine and serum were determined as described in Section 2. Each bar represents the mean value of samples from four experiments in different groups of five mice. Statistical differences between the treatments were (*P* > 0.01).

In Figure 3 is shown the kinetics of cytokines present in urine. The levels of IL-4 present in urine from groups of animals treated with *Cdt* were significantly lower when compared with those obtained from control groups (*P* < 0.001). IL-5 levels peaked after 15 min of *Cdt* administration, decaying thereafter (Figure 3). The highest levels of IL-6 present in urine from mice injected with 1 LD_50_ *Cdt* were observed after 30 min, decaying thereafter (Figure 3). The levels of TNF started to appear after 30 min in urine from mice injected with *Cdt* up to 2 hs. The concentrations of TNF present in urine were significantly higher than those obtained from urine from control group (*P* < 0.0001). Highest levels of IFN-*γ* in urine were observed 30 min after *Cdt* administration, decaying thereafter (Figure 3). The levels of IL-10 in urine started to appear after 30 min up to 4 hs, decaying thereafter. The levels of this cytokine were significantly higher than those obtained from control mice (*P* < 0.0001) (Figure 3). 

**Figure 4:**
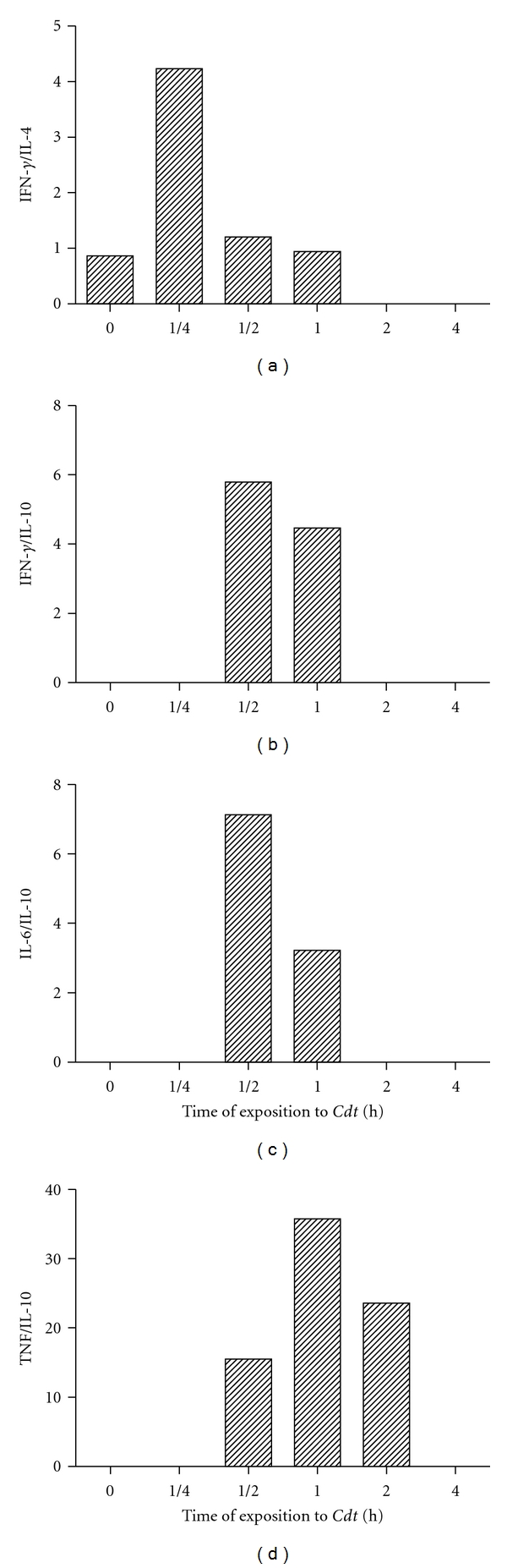
Balance pro-/anti-inflammatory. The urine levels of cytokines were determined as described in Section 2. The ratios IFN-*γ* /IL-4, IFN-*γ*/IL-10, IL-6/IL-10 and TNF/IL-10 represent the values of samples from two experiments in different groups of five mice.

An imbalance of immune mediators together with an inappropriate enhancement of proinflammatory cytokines or even reduction of anti-inflammatory mediators has been recognized to play an important role in the pathophysiology of renal dysfunction. The ratios of pro-/anti-inflammatory were calculated. The IFN-*γ*/IL-4, IFN-*γ*/IL-10, and IL-6/IL-10 ratios increased gradually, reaching their highest at 15 and 30 min, respectively, and decaying thereafter (Figure 4). The highest TNF/IL-10 ratios were observed at 1 h after *Cdt* administration (Figure 4).

**Figure 5:**
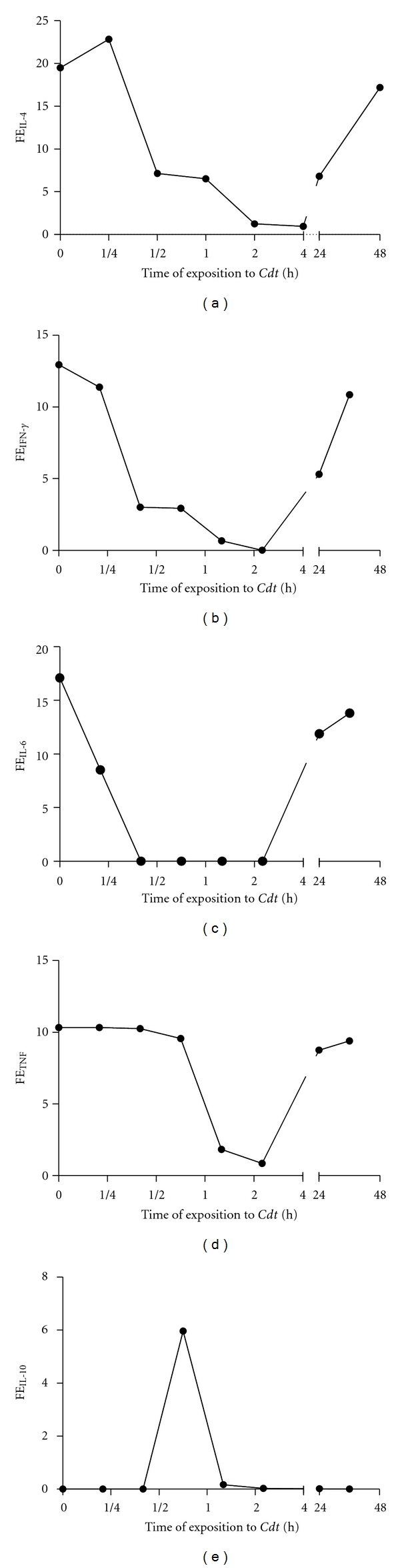
Percentage of fractional excretion of cytokines. Groups of BALB/c female mice were injected i.p. with 1 LD_50_ of *Cdt* for different times as above described. The percentage of fractional excretion was determined as described in Section 2. Each point represents the mean value of samples from four experiments in different groups of five mice. Statistical differences between the treatments were (*P* > 0.01).

The fractional excretion of a substance represents the proportion of the substances excreted in the urine compared with that filtered by the glomeruli. Calculations of fractional excretion help to understand whether increased serum levels of an analytic are due to increased production or decreased excretion. In this study, the fractional excretion of cytokines measured of the percentage of cytokine excreted in the urine versus the cytokine reabsorbed by the kidney was determined. It is measured in terminus of serum and urine cytokine. We previously described the presence of IL-4, IL-5, IL-6, TNF, IFN-*γ*, and IL-10 in serum from female BALB/c mice injected with *Cdt *[24]. The fractional excretion of cytokine was calculated as a part of the evaluation of acute renal failure. The percentage of fractional excretion of IL-4 and IFN-*γ* was reduced in groups of animals treated for 30 min (Figure 5). Fractional excretion of IL-6 was reduced in group of animals treated for 15 min with *Cdt* (Figure 5). The percentage of fractional excretion of TFN-*α* was similar to control up to 1 h post-*Cdt* administration. As shown in Figure 5, the percentage of fractional excretion of TNF-*α* was significantly reduced in groups of animals treated with *Cdt* for 1 up to 2 hs. With respect to the percentage of fractional excretion of IL-10, the high percentage was observed after 1 h of *Cdt* administration, decaying thereafter (Figure 5). Percentages of fractional excretion of all cytokines had recovered considerably after 48 hs. 

### 3.4. Effect of Cdt on Renal Injury

**Figure 6:**
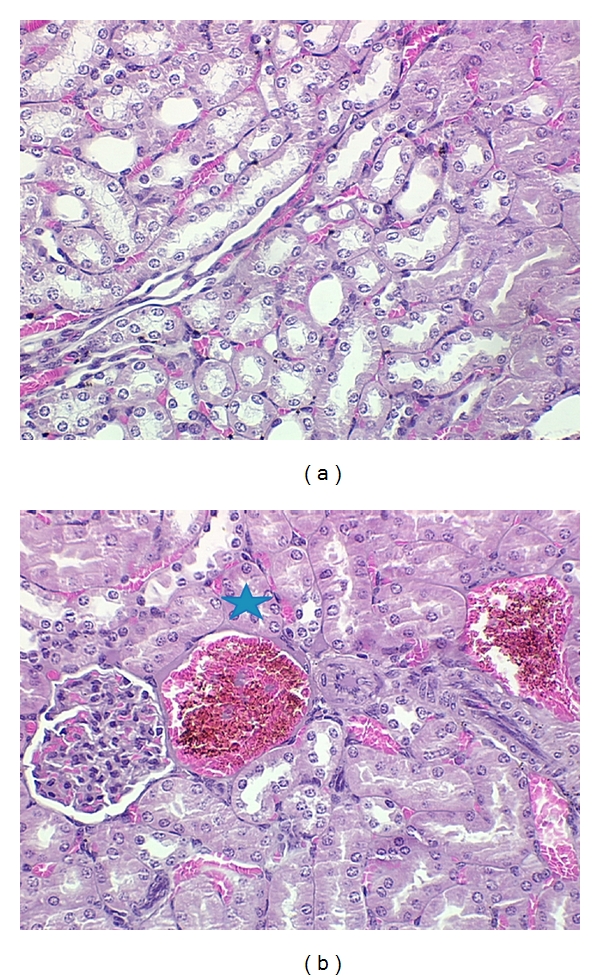
Histological assays. Groups of BALB/c female mice were injected i.p. with 1 LD_50_ of *Cdt* for different times as above described. The levels of cytokines excreted in urine were determined as described in Section 2. Each point represents the mean value of samples from four experiments in different groups of five mice. Statistical differences between the treatments were (*P* > 0.01).

To evaluate the renal injury, groups of mice injected with 1 LD_50_ of *Cdt* for different intervals of time were sacrificed, and the kidneys were histologically examined. The degree of renal injury was graded semiquantitatively in at least 30 cross-sections per mice according to the following characteristics: glomerular lesions, tubular vacuolization, tubular dilation, tubular necrosis, and leukocyte infiltration. Histological evaluation of kidneys from groups of mice injected with *Cdt* is shown in Figure 6. The histological evaluation of kidneys from control groups revealed good preservation (data not shown). *Cdt* injection resulted in diffuse renal changes characterized by severe tubular injury with principal observation in tubular dilation and cast formation throughout the cortex, moderate extra capillary, glomerular hypercellularity, mesangial expansion, and increased glomerular size (Figure 6). The degenerative changes were seen in the proximal tubules at 4 hs after venom injection. These changes considered a loss of proximal brush border, cytoplasmic vacuolation, and in some tubules, degeneration and desquamation of necrotic cells. 

## 4. Discussion

In Brazil, *Crotalus durissus terrificus* is responsible for the majority of accidents among humans. The etiology of *Crotalus *venom-induced acute renal failure in humans and experimental animals is still not completely understood but probably involves a direct action of venom components on renal tubules and renal epithelial cells. The pathogenesis of acute renal failure induced by *Crotalus *venom is multifactorial. It has been associated to direct nephrotoxicity with absence of systemic factors as well as to rhabdomyolysis with the subsequent release of myoglobin from damaged skeletal muscle into serum and urine that are potentially nephrotoxic, leading to acute tubular necrosis [28–31]. 

In order to establish the optimal conditions for renal inflammation-venom interactions, the effects of *Cdt* on levels of proteins, creatinine, and cytokine excreted in urine were studied. This study shows that after *Cdt* injection was observed an increasing of proteins in urine, and these changes were dose dependent of the venom used. Acute renal failure is a clinical entity characterized by a sharp reduction in the glomerular filtration rate and can result from a variety of renal injuries [32]. We previously described the presence of creatinine in serum [24], a combination of blood and urine creatinine levels may be used to calculate a creatinine clearance. In this study we demonstrated that the creatinine clearance was reduced in groups of mice treated with *Cdt* for 2 h with return to the basal levels after 48 h. These results are in accordance with other authors who have described that after envenomation with snake from *Crotalus* and *Bothrops* genera presented reversible alterations in renal function [29, 32, 33]. 

The cytokines play critical roles in initiation and modulation of renal inflammatory responses through their ability to modulate the T helper 1/T helper 2 balances of nephritogenic immune responses. T cells actively participate in renal injury and are mainly with Th1 phenotype and produce TNF-*α* and IFN-*γ* [34–36]. The contribution of Th1 cells in renal pathology has been well described in T-cell-deficient animals, which lack a transcription factor promoting Th1 cells differentiation [37, 38]. The renal inflammation such as glomerulonephritis and tubulo-interstitial nephritis can occur either as an isolated local acute inflammatory reaction or as part a systemic inflammatory disorders, which usually results in interstitial fibrosis, tubular atrophy, and glomerular if not treated or spontaneously repaired. Inflammatory mediators produced and secreted at first hours can induce organ failure and damage. In the present study we observed the acute renal failure in mice injected intraperitoneally with 1 LD_50_ of venom that permitted surgery and physiological measurements in survivor animals. In addition, with this dose of *Cdt*, it was possible to evaluate renal function. At 30 min, envenomation resulted in an elevation in urine IL-6, IFN-*γ*, TNF, and IL-10 concentration. Renal clearances of creatinine were determined at 1-2 h following a *Cdt* administration. The glomerular failure rate decreased significantly 2 h after the venom administration. IL-6 is commonly induced together with other inflammatory cytokines that could affect IL-6 signaling and its biological effects [38]. In accord, IL-1*β*, TNF-*α*, and IL-10 interfere with IL-6 signaling and decreased its anti-inflammatory effects [38]. Therefore, the function of IL-6 as a pro- or anti-inflammatory cytokine is also related to the presence or absence of other inflammatory cytokines. Under the conditions used in the present study, we observed that the injection with *Cdt* may alter cytokine production. The levels of IL-6 and IFN-*γ* present in urine were decreasing in a dose-dependent manner. By contrast, the highest levels of IL-4, IL-5, and IL-10 were obtained in groups of mice injected with 0.5 LD_50_ of* Cdt*. The highest levels of TNF were observed in groups of mice injected with 1 LD_50_. 

Studies aimed to evaluate the role of the kidney in clearance of the inflammatory cytokines of septic patients reveal that the kidney removes some proinflammatory cytokines from plasma at the onset of the disease as long as diuresis is maintained [39]. However, in advanced sepsis characterized by acute renal and oliguria, the fractional excretion of several inflammatory cytokines drops, and, as a consequence, their plasma concentration rises [40]. In humans, the role of the kidney in controlling cytokines homeostasis is well established [40]. The kidney preferentially filters smaller proinflammatory molecules such as <20 kDa and less readily filters the larger anti-inflammatory cytokines that present >20 kDa and soluble cytokines receptors [41]. In this study we show that, among cytokines, TNF-*α* and IL-10 were secreted at high and low levels in urine, respectively. Moreover, the filtered proinflammatory cytokine, are not excreted intact in the urine but are absorbed in the proximal renal tubules and denatured by intracellular proteolytic mechanisms [42].

The ratios of pro-/anti-inflammatory cytokines indicate the inflammatory status of the cells. The results obtained in this study showed the balance between pro- and anti-inflammatory cytokines in urine, which may represent inflammatory status in envenoming processes up to 4 hours after *Cdt* administration. Based on these findings with low levels of anti-inflammatory cytokines and high levels of proinflammatory cytokines that are associated with increased mortality, it seems evident that the balance between pro- and anti-inflammatory cytokines was associated with organ function, which was evaluated by measuring kidney function. Therefore, creatinine levels in blood and urine may be used to calculate the creatinine clearance which reflects the glomerular filtration rate that is clinically important because it is a measurement of renal function. Obstruction of the upper urinary tract has deleterious effects on the kidney and is an important cause of renal insufficiency in humans. In this study the histological analysis of kidneys from envenomated mice revealed that rupture and desquamation of renal tubule epithelium into the tubule lumen could contribute to proteinuria. Changes initially observed in renal function are reversible; however, with sustained injury these histological alterations translate into a permanent loss in renal function [43]. The detection of *B. alternatus* venom in glomerular and in proximal and distal renal tubules agreed with the morphological and histological damage caused by the venom in these anatomical regions and indicated that there was a close correlation between the sites of venom localization and subsequent damage [33]. 

In conclusion, our results demonstrate that, in collected urine from mice injected with *Cdt*, the levels of cytokines were decreased when compared to those in control groups. The cellular localization of the venom and its kinetics in renal tissue agreed with various venom-induced morphological and functional alterations.

## Figures and Tables

**Table 1:** Time course of protein in urine and creatinine clearance.

Time of exposition to *Cdt *	Protein	Creatinine clearance
0	180 ± 9	48.48 ± 1.11
1/4	220 ± 12^b^	3.73 ± 0.11^b^
1/2	300 ± 13^b^	3.47 ± 0.97^b^
1	350 ± 18^b^	3.67 ± 0.11^b^
2	330 ± 15^b^	3.62 ± 0.10^b^
4	300 ± 10^b^	7.45 ± 0.23^b^
6	290 ± 12^b^	13.88 ± 0.36^b^
24	260 ± 16^a^	40.00 ± 1.16^b^
48	240 ± 10^a^	39.80 ± 1.31^b^

Groups of BALB/c female mice were injected i.p. with 1 LD_50_ of *Cdt *for different times as described in Section 2. The presence of protein in urine was determined by measuring the albumin. The creatinine clearance in urine was determined as above described. ^a^Not significant and ^b^ *P* < 0.005.
